# Functional analysis of the *Drosophila* RhoGAP Cv-c protein and its equivalence to the human DLC3 and DLC1 proteins

**DOI:** 10.1038/s41598-018-22794-9

**Published:** 2018-03-15

**Authors:** Sol Sotillos, Mario Aguilar-Aragon, James Castelli-Gair Hombría

**Affiliations:** 1 CABD (CSIC/JA/Univ. Pablo de Olavide), Seville, Spain; 2 Present Address: The Francis Crick Institute, London, UK

## Abstract

RhoGAP proteins control the precise regulation of the ubiquitous small RhoGTPases. The *Drosophila* Crossveinless-c (Cv-c) RhoGAP is homologous to the human tumour suppressor proteins Deleted in Liver Cancer 1–3 (DLC1-3) sharing an identical arrangement of SAM, GAP and START protein domains. Here we analyse in *Drosophila* the requirement of each Cv-c domain to its function and cellular localization. We show that the basolateral membrane association of Cv-c is key for its epithelial function and find that the GAP domain targeted to the membrane can perform its RhoGAP activity independently of the rest of the protein, implying the SAM and START domains perform regulatory roles. We propose the SAM domain has a repressor effect over the GAP domain that is counteracted by the START domain, while the basolateral localization is mediated by a central, non-conserved Cv-c region. We find that DLC3 and Cv-c expression in the *Drosophila* ectoderm cause identical effects. In contrast, DLC1 is inactive but becomes functional if the central non-conserved DLC1 domain is substituted for that of Cv-c. Thus, these RhoGAP proteins are functionally equivalent, opening up the use of *Drosophila* as an *in vivo* model to analyse pharmacologically and genetically the human DLC proteins.

## Introduction

The Rho GTPases cycle between an active state, when bound to GTP, and inactive one, when bound to GDP. Active Rho GTPases control multiple cellular aspects including actin cytoskeleton organization, microtubule dynamics, cell adhesion, cell polarity, endocytosis, progression through cell cycle, differentiation and gene transcription (Reviewed in)^1–5^. Such functional diversity for a ubiquitously expressed single regulator requires the tight spatial and temporal control of its activity. There are two main classes of Rho regulators controlling the cycle between GTP and GDP: the Guanine nucleotide Exchange Factors (GEF) and the GTPase Activating Proteins (GAP)^6^. GEFs activate GTPases by displacing the GDP nucleotide, allowing Rho to bind GTP. GAPs inactivate the Rho-GTP by enhancing Rho’s low intrinsic GTPase activity resulting in Rho-GDP. There are more GEF and GAP regulators than Rho GTPases and this is thought to be fundamental for controlling their localized cellular activity.

All RhoGAP proteins contain a GAP domain consisting of nine α-helices with a tightly conserved catalytic arginine residue required to accelerate Rho GTP hydrolysis^7,8^. The combination of diverse protein domains to the GAP domain confers specific functions to the different RhoGAP proteins. The *Drosophila crossveinless-c* (*cv-c*) gene encodes a RhoGAP protein required for the Actin reorganisation during morphogenesis^9^. Cv-c is homologous to the human Deleted in liver cancer 1, 2 and 3 [DLC1, DLC2 and DLC3] proteins^10^ with both Cv-c and DLC being able to enhance the weak constitutive GTPase activity of Rho leading to its inactivation^11,12^. Loss of DLC1-3 expression is observed in many human cancers, and the restoration of DLC1 expression leads to the inhibition of tumour growth *in vitro*, indicating that the DLC proteins are tumour suppressor proteins. Homozygous DLC-1^−/−^ mice knockout embryos die at day 10.5 *post coitum* with defects in the neural tube, brain, heart and placenta^13^. In contrast, DLC-2^−/−^ knockout embryos are viable^14,15^. DLC-2^−/−^ knockout mice do not have an increase in spontaneous cancer development^15^ and neither do DLC-1 heterozygous mutant mice. However DLC-1 knockdown collaborates with Myc and p53 to induce tumours in a transplant mouse model^16^. In *Drosophila, cv-c* mutation leads to various morphogenetic abnormalities including defects in midgut constriction, head involution, salivary glands, trachea and posterior spiracle invagination, dorsal closure and Malpighian tubule formation^9^. Analysis of Cv-cGFP fusion proteins revealed that Cv-c associates to the basolateral membrane of ectodermal epithelial cells in an opposing localisation to that of two apical RhoGEF activators^17^ suggesting that both Cv-c enzymatic activity and its subcellular localisation are fundamental for the function of this class of RhoGAP proteins.

Comparison of the Cv-c and DLC RhoGAP sequences reveal they are large proteins with three conserved domains: the GAP domain involved in RhoGTP binding, a protein-protein interaction SAM domain and a lipid binding START domain^9^. These domains, organized in the same order, are also present in the human DLC1, DLC2 and DLC3 proteins suggesting that they are all functional homologs. In this work, we investigate the functional requirement of the different Cv-c protein domains, finding which are required for Rho regulation and which for the correct subcellular localization. We find that the DLC3 human homolog, which in vertebrate cells localizes at the adherens junctions^18^, associates to the basolateral membrane of *Drosophila* epidermal cells and behaves as Cv-c. DLC1 can function efficiently in *Drosophila* only if fused to the *Drosophila* subcellular localization domain.

## Results

**Figure 1:**
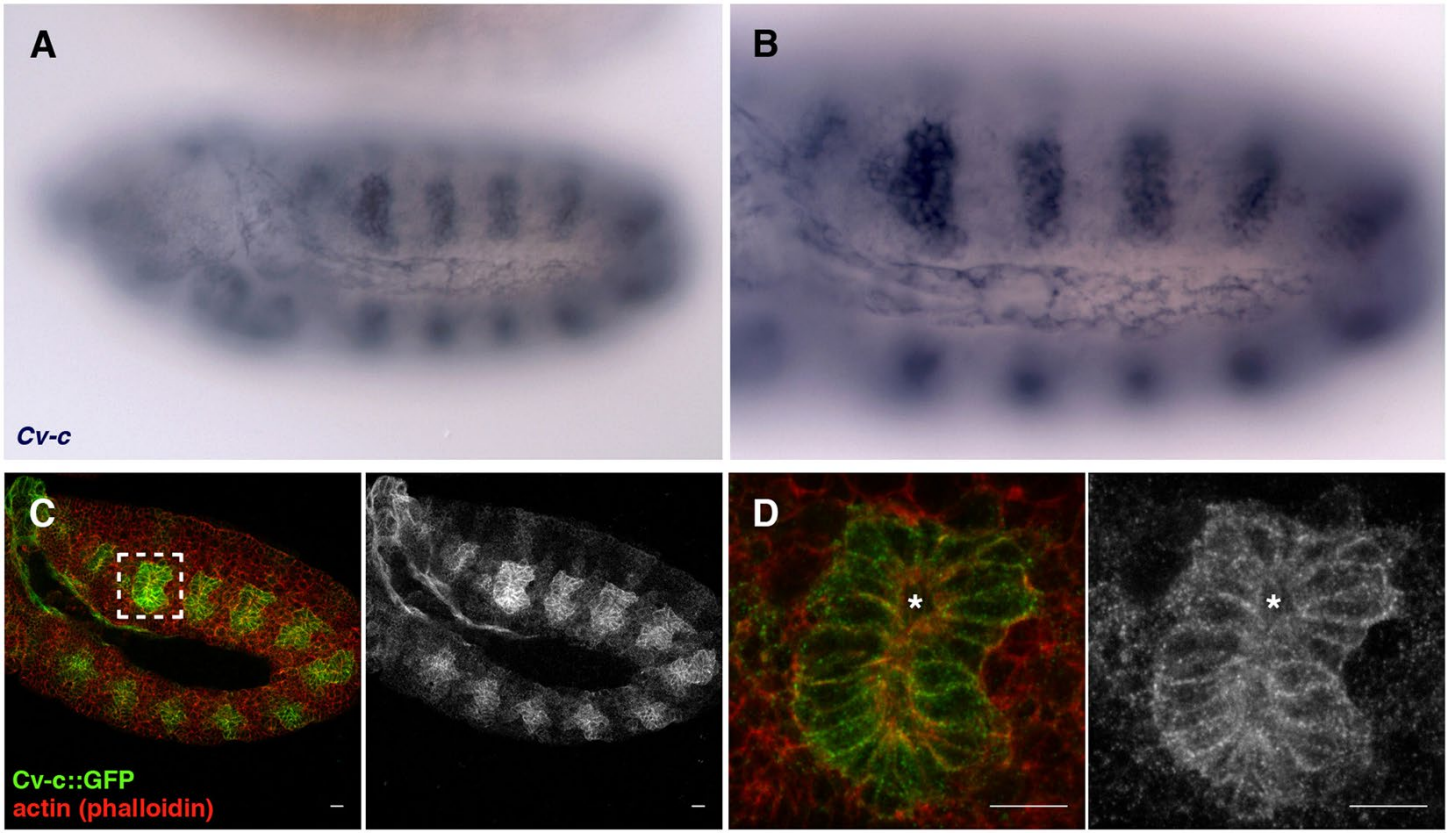
Expression and subcellular distribution of a Cv-c trapped isoform. (**A**) RNA *in situ* showing *cv-c* expression at stage 11 of embryogenesis. (**A**) close-up is shown in (**B**). (**C**–**D**) *Mi{MIC}cv-c*^*MI00245-GFSTF.0*^ st11 embryo double stained for GFP (green) and phalloidin (red) showing that the trapped Cv-c isoform is expressed in the same pattern as the *cv-c* transcript. (**D**) Close-up of the inset in C showing the tagged isoform localisation at the baso-lateral cortex of the invaginating tracheal pit cells (the apical side is facing the lumen, marked by an asterisk).

Based on the localization of ectopically expressed GFP tagged proteins, Cv-c has been reported to associate to the basolateral membrane of epithelial ectodermal cells^17^. To find if Cv-c expressed at the endogenous protein levels also localizes basolaterally, we studied the TRAP line *Mi{MIC}cv-c*^*MI00245*^ where a GFP sequence flanked by a splicing acceptor and a splicing donor is inserted in the intron of one of the Cv-c long isoforms^19^. The resulting “trapped” isoform is expressed in the same pattern as the *cv-c* transcripts (compare Fig. 1A–C) and reveals a basolateral cortex subcellular localization (Fig. 1D), confirming that the ectopically expressed Cv-c-GFP protein reports the correct cellular localization.

### Functional analysis of Cv-c domains

**Figure 2:**
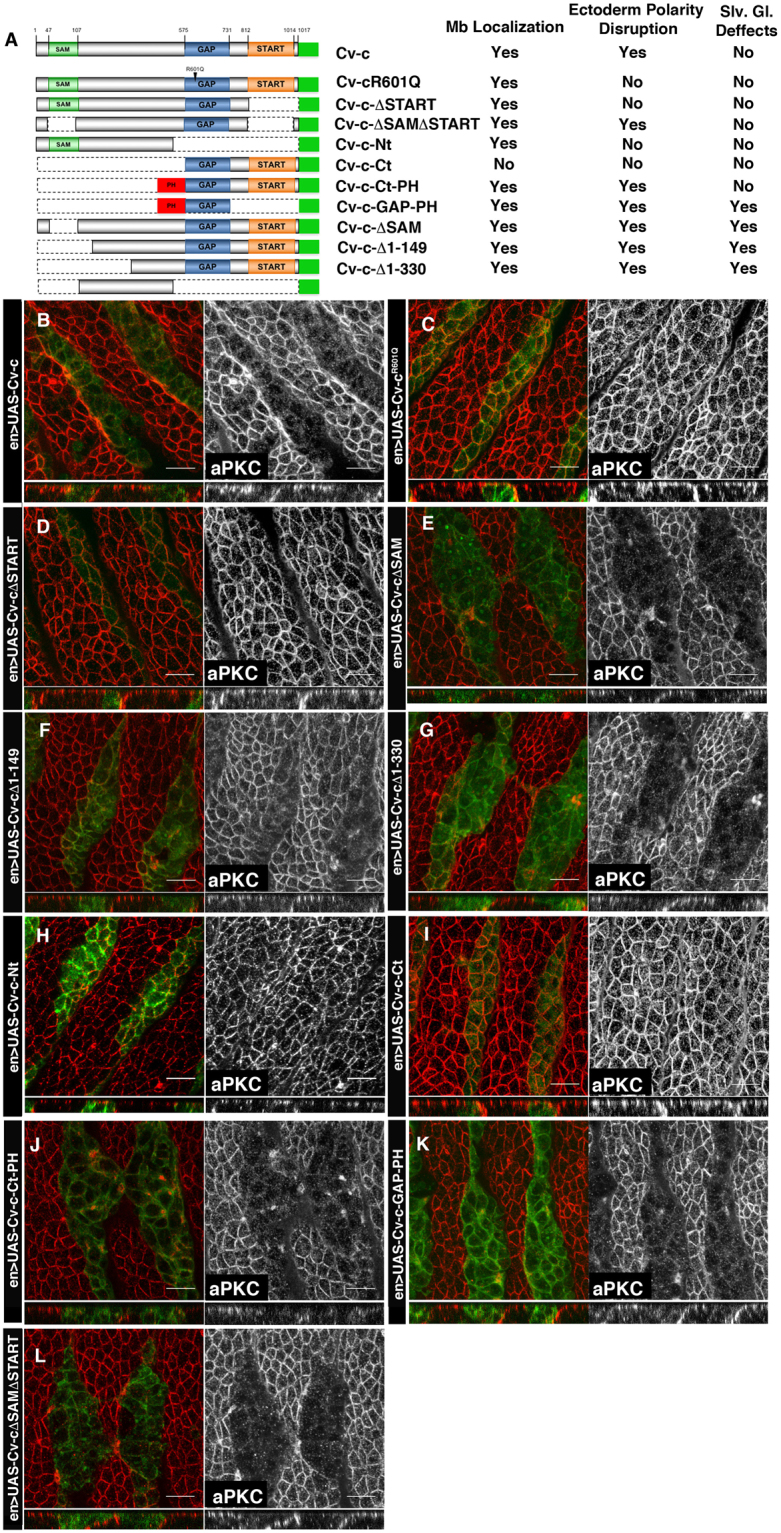
Functional dissection of Cv-c protein. (**A**) Left, Cv-c variants used in this study. All variants were C-terminal tagged to venus-GFP (solid green box). The conserved SAM, GAP and START domains are represented as green, blue and orange boxes. The heterologous PH membrane localization domain is represented in red. The table on the right summarizes the membrane (Mb) localization of Cv-c variants, their ectodermal polarity disruption activity and salivary glands (Slv. Gl.) defects. (**B**–**L**) Effect of Cv-c variant’s expression on aPKC membrane localization. *UAS-cv-c-GFP* variants were expressed in the posterior compartment of each segment with an *en-Gal4* driver line, allowing to compare the behaviour of cells expressing Cv-c with their non-expressing neighbours. aPKC appears grey on the right panels, or red on the left panels. Green labels GFP. (**B**) Full length Cv-c overexpression eliminates aPKC from the apical membrane of the epithelial cells. (**C**) Mutation of a catalytic Arginine in the GAP domain abolishes Cv-c activity. (**D**) The START domain is required for Cv-c function. (**E**–**G**) The SAM domain and the N-terminal third of Cv-c are not required for aPKC down-regulation. (**H**) The N terminal half of the protein is inactive. (**I**) The C-terminal half containing the GAP and START domains is not sufficient for Cv-c function, but activity is recovered (**J**) if the C-terminal half is fused to the heterologous PH membrane localization domain. (**K**) Fusing the isolated GAP domain to a PH domain is sufficient to induce the polarity defects. (**L**) A Cv-c protein with the SAM and START domains deleted can still down-regulate aPKC expression. Confocal Z-sections are shown below the panels. Scale bar: 10 µm.

Previously we showed that ectopic expression of wild type *cv-c* in ectodermal cells normally devoid of Cv-c causes a loss of epithelial characteristics and induces cell migration^20^. The loss of cell polarity caused by full length Cv-c-GFP ectopic expression on epithelial cells offers an excellent model to test in whole animals the activity of Cv-c proteins lacking particular domains. To this end, we made UAS- constructs driving Cv-c variants (Fig. 2A) fused to GFP and expressed them in embryos using the *en-Gal4* line. As previously reported, the expression of a wild type Cv-c-GFP tagged protein causes embryonic death due to apico-basal epithelial polarity disruption that can be detected by the loss of aPKC protein from the subapical epithelial region (Fig. 2B). No effects on embryo viability or polarity are observed after expression of the Cv-c^R601Q^-GFP protein where a single conserved catalytic arginine in the GAP domain is mutated to glutamine, showing that the loss of polarity requires Cv-c’s GAP function^20^ (Fig. 2C). We next expressed a Cv-c protein lacking the START domain (Cv-cΔSTART-GFP, Fig. 2D) and found this to be inactive in the ectoderm, indicating that in the Cv-c protein context the GAP activity requires the presence of the START domain. In contrast, expression of a Cv-c fragment lacking the SAM domain (Cv-c-ΔSAM-GFP, Fig. 2E) does not abolish Cv-c function, and the same is true for proteins deleting the N-terminal 149 a.a. (Cv-cΔ1-149-GFP, Fig. 2F) or 330 a.a. (Cv-cΔ1-330-GFP, Fig. 2G) indicating that the SAM domain and N-terminal third of the protein are not required for Cv-c activity. Moreover, expression of the N-terminal half of the protein (Cv-c-Nt-GFP) does not have any effects on polarity nor viability (Fig. 2H). Finally, we expressed the C-terminal part of the protein (Cv-c-Ct-GFP) that exclusively contains the GAP and START domains and found that this fragment is inactive (Fig. 2I), even when using the stronger driver line *hh-Gal4*, which is expressed in a similar pattern (Supplementary Fig. 1). These results suggest a functional requirement of the non-conserved central region of the protein localized between a.a. position 330 and the GAP domain.

### Regulation of Cv-c basolateral localization

The lack of function of the inactive Cv-c fragments could be caused either by an intrinsic lack of enzymatic activity that would make the resulting protein unable to regulate Rho1, or to a problem with the protein’s subcellular localization that would not allow the Cv-c protein fragment to interact with the membrane bound Rho1-GTP. To distinguish between these alternatives, we analysed the subcellular localization of the Cv-c variants in the large polyploid salivary gland cells. Salivary gland development requires the endogenous Cv-c^21^ and therefore these cells should have all the necessary factors required for Cv-c localization.

**Figure 3:**
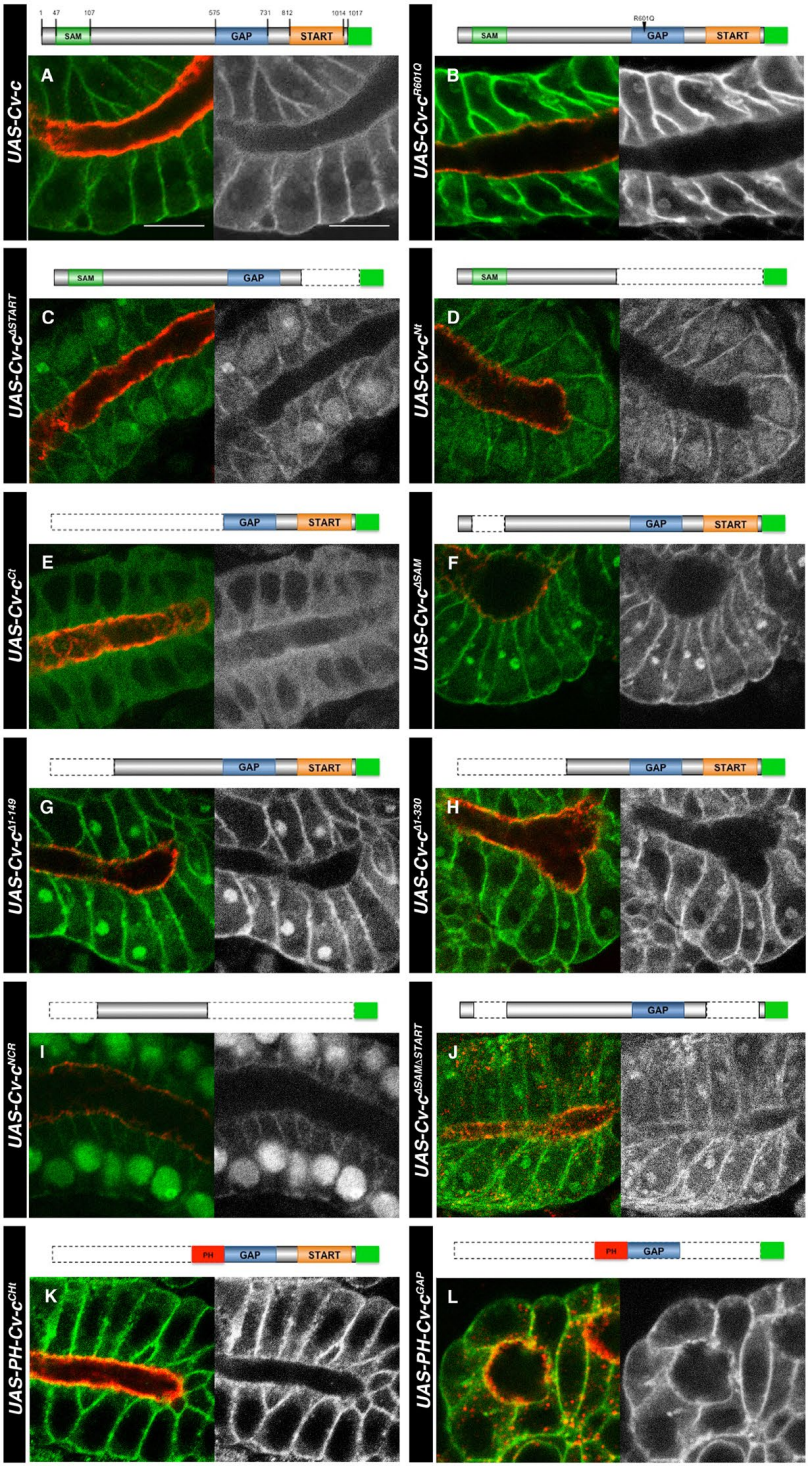
Subcellular localization of Cv-c variants in embryonic salivary gland cells. (**A**–**L**) Confocal sections of the sac-like salivary gland epithelium. The central apical lumen is labelled with aPKC in red. Expressed Cv-c-GFP variants are labelled in green (or grey in right panels). (**A**) Cv-c associates to the basolateral membrane. Cv-c basolateral distribution is unaffected by mutations abolishing the GAP activity (**B**). Cv-c still localizes to the membrane after deleting the START (**C**) or the SAM domains (**F**), although in both cases the cytoplasmic levels are increased. (**D**) The N-terminal half of Cv-c localizes to the basolateral membrane, whereas (**E**) the C-terminal half containing the GAP and START domains remains in the cytoplasm. (**G**–**H**) The 330 N-terminal Cv-c amino acids are dispensable for basolateral membrane localization. (**I**) The central non-conserved Cv-c region localizes GFP to the basolateral membrane although for unknown reasons much of it accumulates in the nucleus. (**J**) A Cv-c protein lacking both the SAM and START domains localizes to the basolateral membrane. (**K**) The C-terminal GAP and START containing half of the protein can be relocated from the cytoplasm to the membrane by fusing it to a heterologous PH domain (compare to **E**). The isolated GAP domain fused to a heterologous PH domain also localizes to the membrane (**L**). Note that absence of the START domain always results in lower levels of membrane associated protein due to much of the protein being shuttled to the nucleus. Scale bar: 10 µm. All panels are at the same magnification.

Cv-c-GFP localizes to the basolateral cortex of the salivary glands (Fig. 3A) and the same is true for the Cv-c^R601Q^-GFP mutated protein (Fig. 3B and Supplementary Fig. 2) indicating that the GAP function is not required for Cv-c localization. Similarly, deletion of the START domain does not abolish basolateral localization although there is a decrease in protein levels as detected by GFP staining intensity (Fig. 3C). Subdivision of the protein in two halves shows that the N-terminal half associates to the basolateral membrane (Fig. 3D) while the C-terminal domain containing the START and GAP domains remains in the cytoplasm (Fig. 3E). Cv-c proteins with deletions of the N-terminal third of the protein (ΔSAM, Δ1-149 or Δ1-330, Fig. 3F–H respectively) still associate to the basolateral membrane. These data suggest that Cv-c’s basolateral localization in the epidermis is directed by the central non-conserved region (dNCR). In agreement, fusion of the 150–510 a.a. dNCR to GFP associates to the basolateral membrane (Fig. 3I). The low levels of membrane localization observed in the dNCR may indicate that other protein domains prevent Cv-c’s shuttling to other cellular compartments. Note that, although more difficult to observe due to the smaller cell size, all protein variants localising to the membrane on the salivary glands also localise to the membrane in the ectoderm (compare Figs 2 and 3).

### Cv-c function requires basolateral localization

The above experiments suggest that the C-terminal half containing the GAP and START domains may be inactive due to its inability to associate to the basolateral membrane. To test this, we made constructs where the Cv-c C-terminal half is fused to heterologous protein domains directing membrane localization. Fusion of the inactive Cv-c-Cterm-GFP to a Pleckstrin homology (PH) domain of the Phospholipase C δ protein^22^ restores membrane localization (Fig. 3K) and functionality as shown by the downregulation of E-Cad, aPKC and Crb and the reappearance of polarity defects (Fig. 2J).

To test if the direct localization of Cv-c’s GAP domain to the membrane would be enough to cause the observed effects, we expressed a GAP-GFP-PH construct. In these conditions, the GAP domain does localize to the membrane (Fig. 3L) and causes polarity disruption (Fig. 2K). The capacity of the GAP domain to induce the full phenotype suggests that it is the only enzymatic domain of the protein and that the rest of the protein domains are required to regulate its function. Interestingly, the isolated membrane tagged GAP domain seems to maintain its small GTPase specificity as shown by the rescue of the PH-GAP effects by an activated form of Rho1 but not by an activated Rac form (Supplementary Fig. 3). The unregulated character of the isolated GAP domain can explain the observation that when expressed on the salivary glands it causes strong defects that are not seen when both the GAP and START domains are present, indicating that the START domain confers some regulatory function to the GAP domain (compare Fig. 3K–L). Morphological salivary gland aberrations are also observed when the SAM domain is missing, suggesting these constructs are hyperactive (Fig. 3F–H). Although we cannot discard some tissue specificity differences between the salivary gland and the ectodermal cells, putting both results together, we propose that Cv-c’s efficient function requires the localisation of the GAP domain to the membrane where it regulates Rho1 activity. Membrane localisation is regulated by the central non-conserved domain while the SAM and START domains have regulatory roles.

### Cv-c domains function in the Malpighian tubules

**Figure 4:**
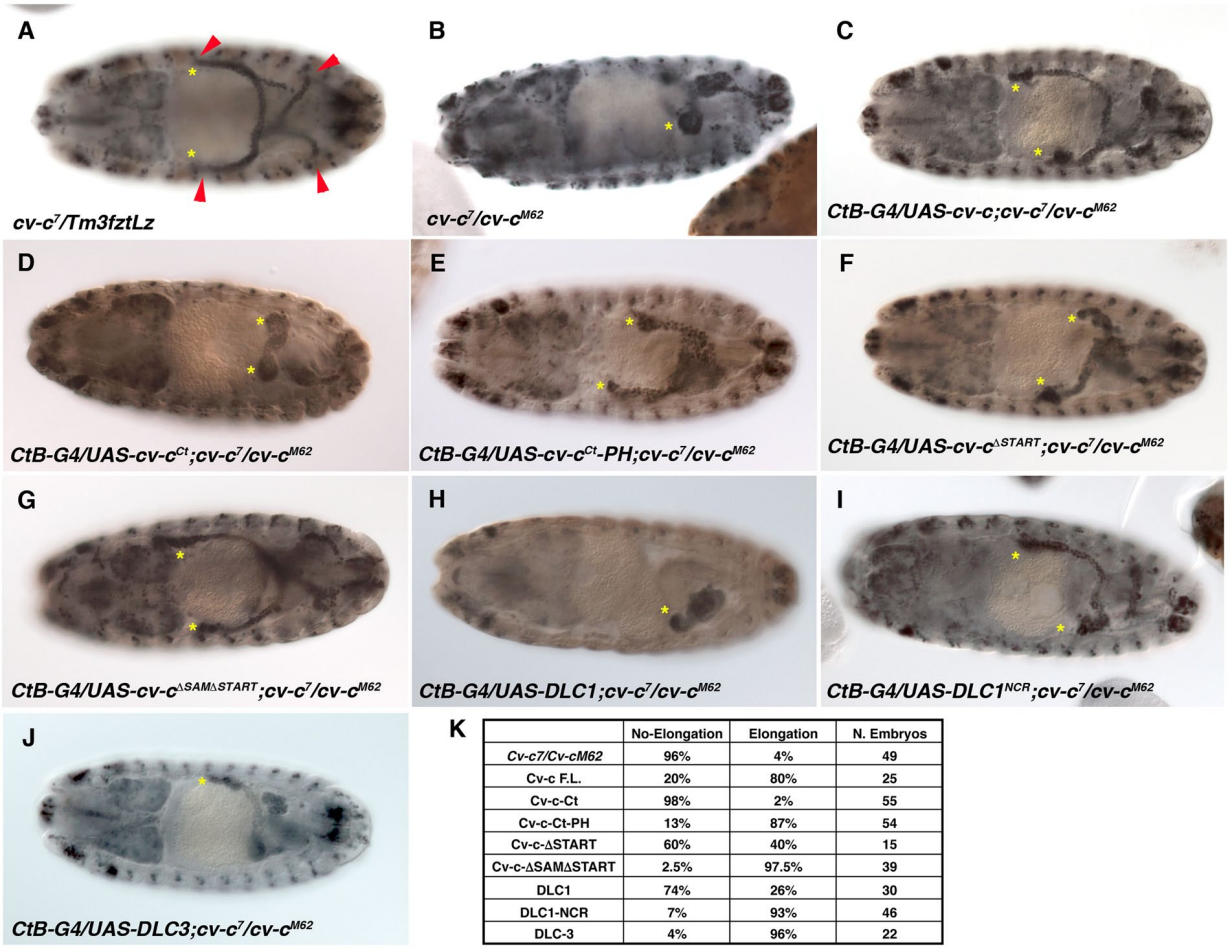
Malpighian tubule elongation rescue by Cv-c/DLC variants. Stage 15–16 *Drosophila* embryos stained with anti-cut to label the Malpighian tubules (asterisks mark anterior Malpighian tubules). (**A**) Normal extended Malpighian tubules formed in heterozygous *cv-c*^7^/+ embryos (red arrowheads). (**B**) Malpighian tubules fail to elongate in mutant *cv-c*^7^*/ cv-c*^*M62*^ embryos. (**C**–**J**) Expression of different Cv-c/DLC variants with the Malpighian specific driver *CtB-Gal4* to test their rescuing capacity. Tubule elongation in mutant *cv-c*^*7*^*/ cv-c*^*M62*^ embryos can be partially rescued by expressing Cv-c-GFP (**C**). The Cv-c-Ct-GFP construct cannot rescue elongation (**D**) unless the membrane tagging PH domain is added (Cv-c-Ct-PH-GFP, **E**). Cv-c-ΔSTART-GFP can also rescue a fraction of the homozygous embryos (**F**), but rescue is more penetrant in Cv-c-ΔSAMΔSTART-GFP where both the SAM and START domains are deleted (**G**). Human Myc-DLC1 shows a weak rescue (**H**) that is increased when the central non-conserved region is substituted by the *Drosophila* Cv-c NCR (**I**). Mild rescue can be observed in most Myc-DLC3 embryos (**J**). (**K**) Quantification of Malpighian tubule rescue in *cv-c*^*7*^*/cv-c*^*M62*^ mutant embryos. Note that the apparent better rescue capacity of the Myc-DLC1-NCR and Myc-DLC3 compared to full length Cv-c-GFP may be due to the different tags used.

Cv-c is expressed in other tissues besides the ectoderm. To test if the Cv-c constructs we tested in the ectoderm function similarly in other organs, we analysed the Malpighian tubules. The Malpighian tubules are four elongated tubular organs that act as insect kidneys (Fig. 4A, arrowheads). In *cv-c* null mutant embryos, the Malpighian tubules do not elongate (Fig. 4B) a defect that can be partially rescued by expressing UAS-Cv-c in the tubules with the *CtB-Gal4* line^9^ (Fig. 4C, K see table for elongation rescue quantification). Testing different deletion constructs we observe that tubule defects cannot be rescued by Cv-c Ct expression, whilst they are rescued by expression of the membrane bound Cv-c Ct-PH (compare Fig. 4D,E) reinforcing that, as we observe in the ectoderm, membrane association of Cv-c is required for its efficient function. In contrast to the ectoderm, expression of a Cv-cΔSTART protein provides some Malpighian tubule rescue (Fig. 4F) although this is more penetrant when expressing a Cv-cΔSAMΔSTART construct (Fig. 4G), which also localizes in the membrane (Fig. 3J) and interferes with polarity in the ectoderm (Fig. 2L). Thus, in the Malpighian tubules Cv-cΔSTART has some RhoGAP activity that can be potentiated by the deletion of the SAM domain. Although these results reveal some minor tissue specific differences, the overall function of the Cv-c domains is similar in different tissues and reinforce that membrane association of Cv-c is required for its efficient function.

### Functional capacity of the human DLC homologs in *Drosophila*

**Figure 5:**
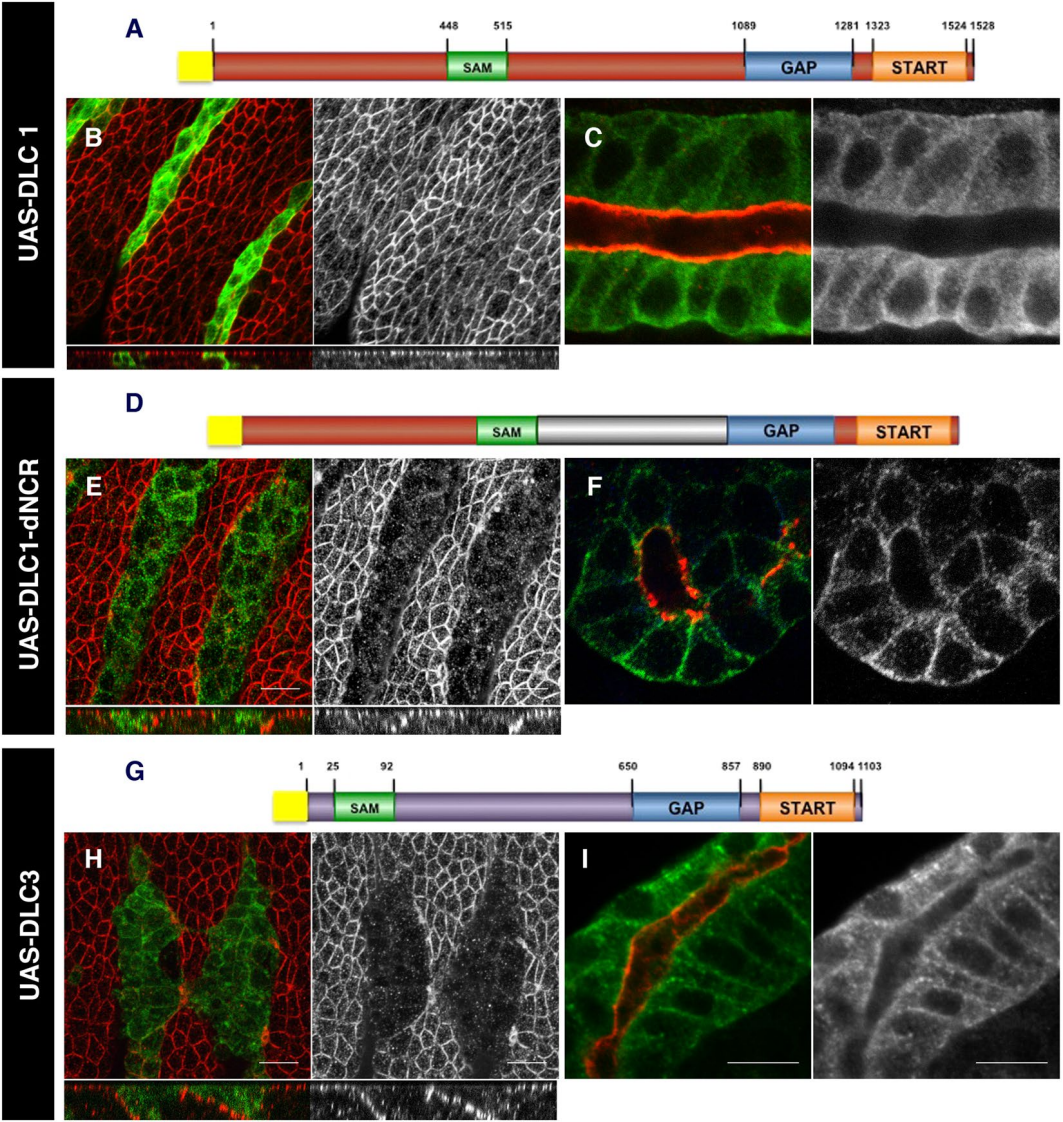
DLC function in *Drosophila*. Expression of DLC1 and DLC3 proteins in the epidermis (**B**,**E** and **H**) and the salivary glands (**C**,**F** and **I**) of *Drosophila* embryos. (**A**–**C**) Expression of a Myc tagged DLC1 (**A**) does not interfere with apical polarity in epithelial cells (**B**) and localizes in the cytosol (**C**). (**D**–**F**) Substitution of the human non-conserved central region with the non-conserved central region of *Drosophila* (dNCR, grey in **D**) confers activity to the DLC1 chimeric protein (**E**) and localizes to the basolateral membrane (**F**). (**G**–**I**) Expression of a Myc tagged DLC3 protein (**G**) causes apical polarity defects (**H**) and the protein can be detected at the basolateral membrane (**I**). (**B**,**C**,**E**,**F**, and **H**,**I**) DLC proteins are detected with anti-myc (green); aPKC is shown in red. Above the panels we show a scheme of the DLC variant expressed with the Myc-tag represented as a yellow box and the conserved SAM, GAP and START domains as green, blue and orange boxes. Non-conserved regions (NCR) are represented in grey for Cv-c, brown for DLC1 and purple for DLC3. In B,E,H confocal Z-sections are shown below the panels. Scale bar: 10 µm.

Given the sequence conservation between Cv-c and DLC, we tested in our system if the human homologs would have a similar function and thus *Drosophila melanogaster* could serve as an animal model to study DLC. Of the three human homologs, only DLC3 has been described to associate to the membrane, interacting with the adherens junctions and being required for cell junction stability^18^. We found that in embryos expressing a *UAS-Myc-tagged DLC-3* construct with *en-Gal4* (Fig. 5G), DLC3 associates to the basolateral membrane and causes defects similar to Cv-c expression (Fig. 5H–I) with the expressing cells losing cell polarity markers and becoming migratory as also happens with Cv-c.

We next tested the effect of expressing the human tumour suppressor DLC1 tagged with Myc (Fig. 5A). No defects were observed on the epidermis of DLC1 expressing cells, that present normal shape and polarity protein expression (Fig. 5B and Supplementary Fig. 1C) and survive to adulthood giving rise to healthy flies. DLC1 does not associate to the basolateral membrane in vertebrates, and the analysis of Myc-DLC1 subcellular localization in *Drosophila* shows that the protein is mainly cytoplasmic when expressed epithelial cells (Fig. 5C). To test if the lack of activity is due to its failure to associate to the basolateral membrane, we substituted the DLC1 central NCR for the equivalent *Drosophila* NCR (Fig. 5D, grey fragment). The resulting chimeric protein (DLC1-dNCR) associates to the basolateral membrane in epithelial cells (Fig. 5F) and shows similar phenotypes to those caused by Cv-c expression (Fig. 5E).

We next tested whether the DLC human proteins could replace the endogenous Cv-c function by analysing their capacity to rescue the Malpighian tubule defects in *cv-c* mutant embryos. We find that the Malpighian tubule elongation failure observed in the *cv-c* mutant embryos is reverted in embryos expressing DLC-3 and DLC-1-NCR (Fig. 4I–J). In contrast, DLC-1 only showed a poor rescue (Fig. 4H,K), indicating that, when overexpressed, DLC-1 has some RhoGAP activity that can be potentiated by its membrane association.

The above results demonstrate that the basic Cv-c/DLC protein function is conserved although the localization of some of the vertebrate homologs has diversified allowing the DLC proteins to exert their function in different subcellular locations. These results also show that *Drosophila* can be used as an *in vivo* animal model to isolate regulators and test drugs that modulate DLC function.

## Discussion

Epithelial invagination is mediated in most cases by the constriction of an apical actin cytoskeleton whose polarized formation depends on the differential accumulation of active Rho1 in the apical cortex. The RhoGAP proteins play a fundamental role in the precise temporal and subcellular regulation of the Rho GTPases. In *Drosophila* this can be achieved by the opposing localization of RhoGEF activators to the apical membrane and the basolateral localisation of the RhoGAP Cv-c protein^17^. This arrangement results in apical accumulation of Rho1GTP mediated by GEF activity, while active Rho1GTP is absent from the basolateral membrane due to the RhoGAP function^17^. Given the importance of the precise subcellular localization for Cv-c to control GTPase activity we have used the Gal4 inducible expression system to identify the protein domains required for its localisation and function. Our observation that translocating the GAP domain to the plasma membrane (PH-GAP-GFP) with a heterologous Pleckstrin Homology domain mimics the phenotypes caused by the full length Cv-c-GFP expression, suggests that Cv-c epithelial activity only requires the association of its GAP domain to the membrane. The capacity of activated Rho1V14, but not RacV12, expression to rescue the PH-GAP induced effects, demonstrates that the isolated Cv-c GAP domain maintains its specificity for Rho1. We also found that a central non-conserved region (a.a. 330 to 570) between the SAM and the GAP domains is involved on Cv-c’s endogenous basolateral membrane localization. Fusion of this central domain to venus-GFP can induce GFP basolateral membrane translocation, while a Cv-c variant lacking this central region remains scattered in the cytoplasm. Although not conserved in sequence, the equivalent region in DLC1 is also required for proper localization at the focal adhesions^23,24^ hinting at a functional conservation of these a.a. for subcellular localization.

The functional capacity of a membrane localised GAP domain and of a Cv-c protein lacking both the SAM and START domains (Cv-cΔSAMΔSTART, Figs 2L and 3J) indicates that the main function of SAM and START is regulatory. Analysis of N-terminal deleted constructs show the SAM domain is dispensable for Cv-c GAP activity, as the expression of SAM deleted variants cause the same effects as expression of a full length Cv-c. However, although these constructs are active, the GAP domain seems to have lost some capacity to be regulated by the endogenous controls, as while wild type Cv-c expression does not cause any defects when expressed on the salivary glands (a tissue where Cv-c is endogenously expressed) all constructs lacking the SAM domain lead to abnormal glands, presumably due to their inability to respond to internal negative regulation. In the ectoderm the function of constructs lacking the SAM domain does not differ significantly from that of a construct deleting both the SAM and the START domains (Cv-cΔSAMΔSTART, Fig. 2L). This contrasts with the complete ectoderm loss of function of a Cv-c protein containing the N-terminal domains but lacking the START domain (Cv-cΔSTART, Fig. 2D), which suggests that the START domain is necessary to prevent the repression of GAP function by the SAM domain. Based on the above genetic results, we propose that the SAM domain acts as a repressor module for the Cv-c GAP function and that this repressor activity is modulated by the START domain in the ectoderm and salivary glands (Supplementary Fig. 4). In the Malpighian tubules this regulation may be more complex as shown by the fact that constructs lacking a START domain are not completely inactive. This result suggests that, besides the START domain, another domain or post-translational regulatory mechanism may be regulating the SAM interference.

These *Drosophila* observations agree with published results in other organisms. The observation that the *C. elegans* Cv-c homolog lacks a SAM domain^9^, and that DLC2 isoforms with or without a SAM domain are functional^25^ fits with a model where the SAM domain has a purely regulatory function in all Cv-c/RhoGAP proteins. Similarly, the fact that the START domain is not required for the isolated GAP activity of DLC1^12^ but START deleted DLC1 proteins behave as a complete lack of function despite their correct subcellular localization^12,24^ could be explained if the SAM domain repressed the GAP function in the absence of a START domain. In this view, a part of START domain function would only be required in the presence of the SAM domain, explaining that the isolated GAP domain is active when bound to the membrane. Although future research should investigate in depth the biochemical mechanisms regulating Cv-c/DLC proteins, there are already indications of how this regulation may happen. Published results show that phosphorylation of DLC1 in the region between SAM and GAP domains by CDK5 is required to displace the interaction and inhibitory effect of the SAM domain^26^.

The similarities between Cv-c and the human DLC homologs led us to study the functional capacity of DLC1 and DLC3 in *Drosophila*. When expressed in the ectoderm, DLC1 does not have any function in *Drosophila*. This must be due to its incorrect cellular localization, as a Cv-c-like activity is observed when the DLC1 region between the SAM and GAP domains is substituted for the equivalent non-conserved *Drosophila* region. This chimeric protein causes strong salivary gland defects (Fig. 5D,F) indicating that the *Drosophila* regulatory machinery cannot control the SAM and START domains of DLC1 in the epithelium.

In vertebrate models DLC1 localises mostly to the focal adhesions, while DLC3 localizes to the lateral membrane of epithelial cells where it has been linked to the maintenance of E-Cad and adherens junctions. When expressed in *Drosophila*, DLC3 also localises to the membrane and causes similar effects to Cv-c expression. Moreover, DLC3 is able to rescue the loss of function of *cv-c* in the Malpighian tubules to a similar extent as Cv-c, whilst DLC1 is only fully functional when it contains the Cv-c’s membrane localization domain, confirming DLC3 is more similar to Cv-c than DLC1. However, the slight anomalies caused by DLC3 in the salivary glands suggest this protein is not perfectly regulated in *Drosophila* although we cannot discard that the N-terminal Myc-tag in this construct interferes with the SAM regulation.

Similar to Cv-c, DLC3 membrane localization is independent of the SAM domain or its GAP activity^18^. Membrane recruitment depends on the polarity protein Scribble, which is mediated through the interaction of the PDZ3 domain of Scribble and a PDZ ligand motif at the C terminal region of DLC3^27^. Cv-c also contains a PDZ ligand motif at its C terminal region but it seems not to be sufficient for Cv-c localization since the C terminal half of the protein is unable to locate basolaterally. In accordance, we observe that in *scribble* mutants Cv-c still can localize in the membrane (Supplementary Fig. 5).

In summary, our work demonstrates the functional conservation of the DLC/Cv-c RhoGAP proteins during evolution, underscoring the importance of the specific cellular localization of these Rho regulators. Our data also open up the possibility of using *Drosophila* as an *in vivo* animal model to test DLC function or isolate new regulators.

## Methods

### Fly Stocks and Genetics

UAS-cv-c constructs were expressed with the *en-Gal4* or *hh-Gal4* (posterior ectoderm epithelial compartments), *CtB-Gal4* (Malpighian tubules) and *69B-Gal4* (ectodermal epithelium and salivary glands) driver lines (Flybase). The following lines: *UAS-Rho*^*V14*^ and *UAS-Rac*^*V12*^, *Mi{PT-GFSTF.0}cv-c*^*MI00245-GFSTF.0*^, *scrib*^*j*7*B3*^ (Flybase) were obtained from the Bloomington *Drosophila* Stock Center. *cv-c*^*M62*^*, cv-c*^7^were described in^9^. The recombined *Mi{MIC}cv-c*^*MI00245*^ *scrib*^*j*7*B3*^ alleles were balanced over TM6B vvl risk-LacZ to distinguish the homozygous embryos.

Rescue experiments were performed crossing: females CtB-Gal4/CyO ftzLacZ; cv-c ^M62^/TM3 ftzLacZ with males UAS-X/CyO ftzLacZ; cv-c^7^/TM3 ftzLacZ (where X corresponds to cv-c-GFP, cv-c-ΔSTART-GFP, cv-c-ΔSAMΔSTART-GFP, cv-c-Ct-GFP PH-cv-c-Ct-GFP, Myc-DLC1, Myc-DLC1-NCR or Myc-DLC3).

### Immunohistochemistry

Embryos were fixed 20 minutes in a mix formaldehyde 4%/heptane, after removing the heptane, MetOH was added and the vitelline membrane removed by vigorous shaking.

The following primary antibodies were used: guinea pig anti-Scrib (a gift from D. Bilder), mouse anti-Myc (Cell Signalling 1:500), rabbit anti-GFP (Molecular Probes, 1:300), mouse anti-β-gal (Cappel, 1:1.000), mouse anti-aPKC (Santa Cruz Biotechnology 1:100), anti-Dlg (1:100) from Developmental Studies HybridomaBank, Phalloidin-Rhodamine (Molecular Probes). Secondary antibodies were coupled to Alexa488, Alexa555 or Alexa647 (Molecular Probes).

Images were taken on an SP2-AOBS or an SPE Leica confocal microscopes and processed using FIJI and Adobe Photoshop programs. For the higher resolution images in Suppl. Figure 2, a Zeiss LSM 880 Laser Scanning Microscope with Airyscan was used.

### Constructs

Cv-c variants were generated by PCR using Cv-c venus cDNA clone in pENTER^17^ as a template, and subsequently cloned into pTWV or pTVW (UASt promoter, C-terminal Venus tag was obtained from The Drosophila Gateway Vector Collection, Carnegie Institution of Washington, Baltimore, MD; (www.ciwemb.edu/labs/murphy/Gateway%20vectors.html) using the Gateway technology (Invitrogen). In all constructs GFP was inserted at the C-terminal end as we previously noticed that GFP fusion to the N-terminal domain increases Cv-c activity probably due to interference with the SAM domain regulatory activity.

The oligonucleotides used were (5′-3′):

Cv-c R601-Qfor: GGAATTTTCCAGAAAAGCGGTGGAAAGTCGC

Cv-c R601-Qrev: GCGACTTTCACCGCTTTTCTGGAAAATTCC

Cv-c Nt-for: TCCACTTCGCTGGAGGATCATTAGC

Cv-c Nt-rev: GGCGGCAAATGGCATTCCTCCGG

Cv-c Ct-for: CATGGTGAAGGGGGCGGCCGC

Cv-c Ct-rev: CAGACTCTTCCCTTGGCCGTGCG

Cv-c Δ1-149-for: CATGGTGAAGGGGGCGGCCGC

Cv-c Δ1-149-rev: GGCCTGGAACTGCCGCCTCCGG

Cv-c Δ1-330-for: CATGGTGAAGGGGGCGGCCGC

Cv-c Δ1-330-rev: AAGAATTCATGTCGGCGGGTCAACTGC

Cv-c ΔSAM-for: AGCTTCGATTTCTGCAATCTTGCG

Cv-c ΔSAM-rev: CTGGACCAATCGCACAAACCG

Cv-c ΔSTART-for: CCGCCAGTTGTGAAACTGCATTCC

Cv-c ΔSTART-rev: AGCAAAGTTAAGGGTGGGCGCGCC

To generate the UAS-PH-Cv-c-Ct the PH domain of Phospholipase C ∂ was amplified using the following primers:

PLC-PH-NotI: CTCCGCGGCCGCCCCCTTCACCATGGACTCGGGCC

PLC-PH-EcoRI: AAGAATTCCTGGATGTTGAGCTCCTTCAGG

The PCR fragment was digested with NotI-EcoRI and subcloned in a modified version of pENTER-Cv-c-Ct used to subclone the construct in pTVW vector using the Gateway technology (Invitrogen).

To generate the 150-510 fragment of Cv-c the following primers were used:

Cv-c150-510for: CACCATGCGGCTGGACCAATCGC

Cv-c150-510rev: GGCGGCAAATGGCATTCCTCCGG

Cloned in pENTER-DTOPO to subclone the construct in pTWV vector using the Gateway technology (Invitrogen).

To create the UAS-Myc-DLC1 construct, the human DLC1 was amplified by PCR using DLC1-pCMV-sport6 as a template obtained from the Mammalian Gene Collection (GenBank accession number: BC054511) and subcloned into KpnI-XbaI sites of the pUAST plasmid modified to insert a Myc tag at the N-terminus.

To produce the UAS-Myc-DLC1-dNCR, the N terminal and the C terminal part of UAS-Myc-DLC1 and the non-conserved region of Cv-c-pENTER were amplified by PCR using the following primers:

DLC1-N-for: GGAAGCTTGTGATTGGCTACGGGC

DLC1-N-rev: TTTGTGCGATTGGTCCAGCTTCATCACCGCACATTTG

DLC1-C-for: ATCCTCCAGCGAAGTGGAAAATACACACCTTCTAACAAGC

DLC1-C-rev: TCAAATCTTTCTGATCTGGTTTGCCC

Cv-c NCR-for: AAATGTGCGGTGATGAAGCTGGACCAATCGCAC

Cv-c NCR-rev: GTTAGAAGGTGTGTATTTTCCACTTCGCTGGAGG

A mix of these 3 PCRs was used as a template to amplify the DLC1-dNCR using as primers DLC1-N-for and DLC1-C-rev. The PCR product was cloned in pGEMT (Promega), sequenced and subcloned into ClaI-EcoRI in the modified pUASt-Myc.

To generate UAS-Myc-DLC3 a digestion with EcoRI was performed to isolate DLC3 DNA from the pEGFPC1–DLC3α^18^ construct and the fragment subcloned in pUASt-Myc.

Constructs were injected in *D. melanogaster* by Bestgene (USA) and the *Drosophila* Consolider-Ingenio 2007 transformation platform (Spain).

## Electronic supplementary material

Supplementary information
