# Author Correction: Functional analysis of the *Drosophila* RhoGAP Cv-c protein and its equivalence to the human DLC3 and DLC1 proteins

**DOI:** 10.1038/s41598-020-68551-9

**Published:** 2020-07-07

**Authors:** Sol Sotillos, Mario Aguilar-Aragon, James Castelli-Gair Hombría

**Affiliations:** 1 CABD (CSIC/JA/Univ. Pablo de Olavide), Seville, Spain; 2 Present Address: The Francis Crick Institute, London, UK

Correction to: *Scientific Reports* 10.1038/s41598-018-22794-9, published online 15 March 2018

This Article contains errors.

As a result of an error during figure assembly, Figure 5H is a reverse duplication of Figure 2L. The corrected Figure 5 is shown below as Figure 1.

**Figure 1:**
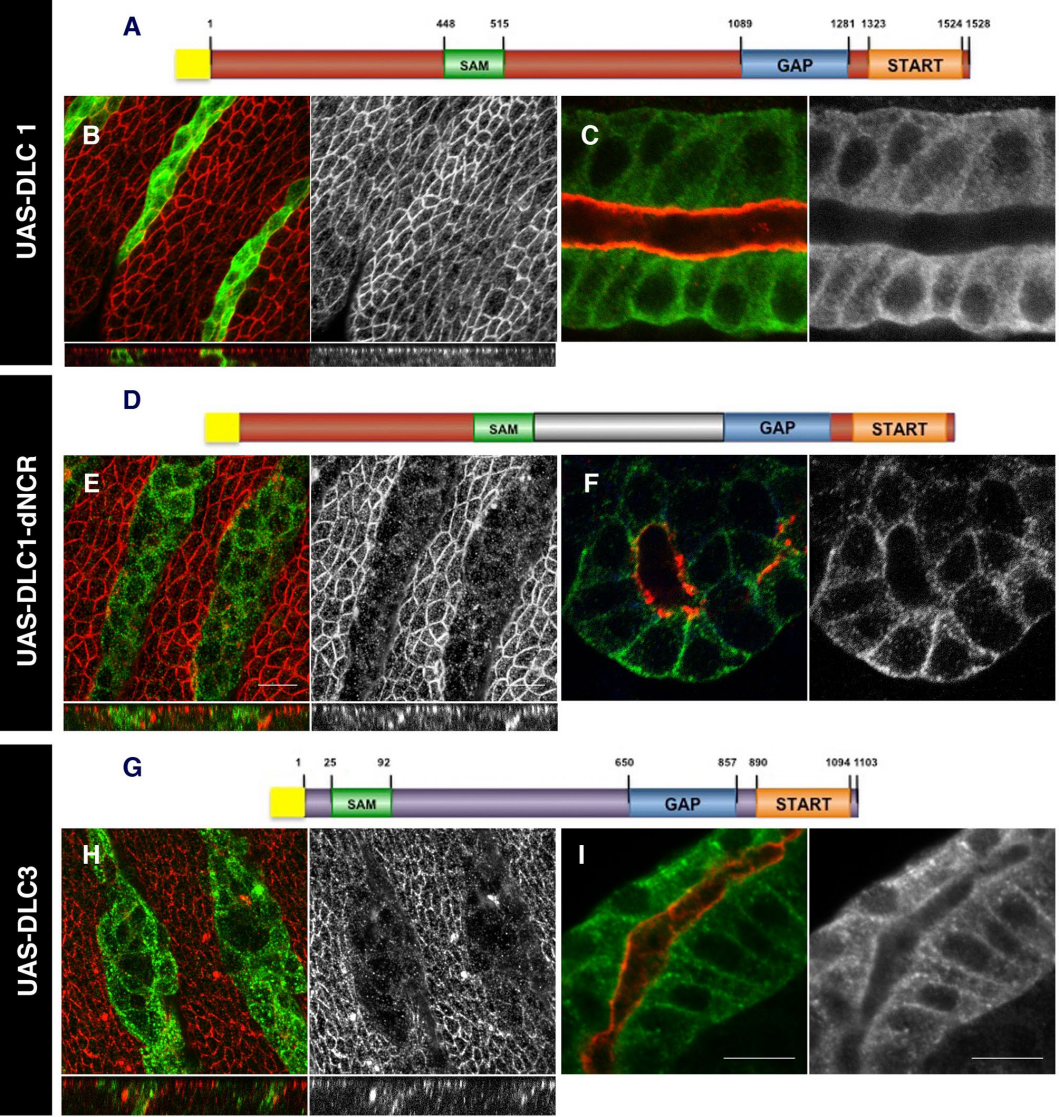
DLC function in *Drosophila*. Expression of DLC1 and DLC3 proteins in the epidermis (**B**,**E** and **H**) and the salivary glands (**C**,**F** and **I**) of *Drosophila* embryos. (**A**–**C**) Expression of a Myc tagged DLC1 (**A**) does not interfere with apical polarity in epithelial cells (**B**) and localizes in the cytosol (**C**). (**D**–**F**) Substitution of the human non-conserved central region with the non-conserved central region of *Drosophila* (dNCR, grey in** D**) confers activity to the DLC1 chimeric protein (**E**) and localizes to the basolateral membrane (**F**). (**G**–**I**) Expression of a Myc tagged DLC3 protein (**G**) causes apical polarity defects (**H**) and the protein can be detected at the basolateral membrane (**I**). (**B**,**C**,**E**,**F**, and **H**,**I**) DLC proteins are detected with anti-myc (green); aPKC is shown in red. Above the panels we show a scheme of the DLC variant expressed with the Myc-tag represented as a yellow box and the conserved SAM, GAP and START domains as green, blue and orange boxes. Non-conserved regions (NCR) are represented in grey for Cv-c, brown for DLC1 and purple for DLC3. In B,E,H confocal Z-sections are shown below the panels. Scale bar: 10 μm.

