# Supplementary material for: Functional analysis of the Drosophila RhoGAP Cv-c protein and its equivalence to the human DLC3 and DLC1 proteins
Source: Sci Rep. 2018 Mar 15;8:4601. doi: 10.1038/s41598-018-22794-9 (PMC5854602; doi:10.1038/s41598-018-22794-9)
Supplement: Supplementary file 1 — Supplementary information [file 41598_2018_22794_MOESM1_ESM.pdf]

Functional analysis of the *Drosophila* RhoGAP Cv-c protein and its equivalence to the human DLC3 and DLC1 proteins

Sol Sotillos[1*], Mario Aguilar-Aragon[1,2] and James Castelli-Gair Hombría[1*]

[1] CABD (CSIC/JA/Univ. Pablo de Olavide). Seville, Spain

[2] Current address The Francis Crick Institute. London, UK.

* Corresponding authors

Correspondence to: jcashom@upo.es; ssotmar@upo.es

Supplementary Figure 1.

Stage 13 *hh-Gal4* embryos expressing in the posterior part of each segment Cv-c-Nt-GFP (A), Cv-c-Ct-GFP (B) or Myc-DLC1. *hh-Gal4* is a stronger driver than en-Gal4 and in these experiments both the Gal4 driver and the UAS constructs were homozygous to test if when expressed at higher levels, these proteins affect polarity. Even in these conditions apical polarity is not affected as shown by the apico-lateral marker aPKC (arrows).

aPKC appears grey on the right panels or red on the left panels. Green cells are labelled in (A-B) with anti GFP or with Myc in (C). Confocal Z-sections are shown below the panels. Scale bar: 10μm.

Supplementary Figure 2. High resolution imaging of Cv-c subcellular localization.

(A) *Drosophila* salivary gland expressing UAS-Cv-c^{R601}-GFP under the control of *69B-Gal4* analysed with high-resolution microscopy. (B) A close-up of the inset in A clearly shows Cv-c-GFP (green) accumulates in the baso-lateral membrane and there is no detectable co-localization with aPKC (red). GFP is green (upper panels) or grey (lower panels) and aPKC red. Scale bar: 10μm.

Supplementary Figure 3. Specificity of the Cv-c GAP domain.

(A-C) *en-Gal4* embryos expressing the GAP domain fused to the PH domain either alone (A) or in combination with *UAS-RhoV14* (B) or *RacV12* (C). Expression of the V14 activated form of Rho rescues the GAP induced polarity phenotypes, while the activated V12 form of Rac does not. aPKC is shown grey (right panels) or red (left panels); GAP-PH-GFP is labelled in green. Confocal Z-sections are shown below the panels. Scale bar: 10μm.

Supplementary Figure 4. Tentative model of Cv-c function regulation.

(A) In the basolateral membrane Cv-c is in an equilibrium between an inactive state, when the SAM domain blocks the GAP function (left) and an active state, when the START domain antagonizes the SAM domain (right) liberating the GAP domain that now can interact with membrane bound Rho1GTP. Cv-c association to the membrane depends on the presence of the Non Conserved Domain (NCR). This equilibrium would be shifted to the active state due to conformational changes induced by Cv-c post-translational modifications or interaction with another protein. This model explains that the deletion of the SAM domain (B) hyperactivates the protein due to lack of negative regulation, and the deletion of the START domain (C) allows the SAM domain to repress GAP activity. In the absence of SAM and START domains (D) Cv-c can function as a RhoGAP although it is less receptive to the endogenous regulatory mechanisms.

Supplementary Figure 5. Cv-c localization in *scribbled* (*scrib*) mutant embryos.

Stage 12 *Mi{MIC}cv-c*$^{MI00245\text{-}GFSTF.0}$ embryos in a wild-type background (A) or recombined with a *scrib*^{j7B3} allele (B) stained against GFP (green), Scribbled (red) and aPKC (blue). Middle panels show a close-up of the eighth abdominal tracheal pit (yellow box) where Cv-c-GFP can be visualized in the membrane of the tracheal cells in both backgrounds. Right panels show Cv-c and Scrib single channels. Scale bars: 100 µm (left) and 10 µm (middle panel).

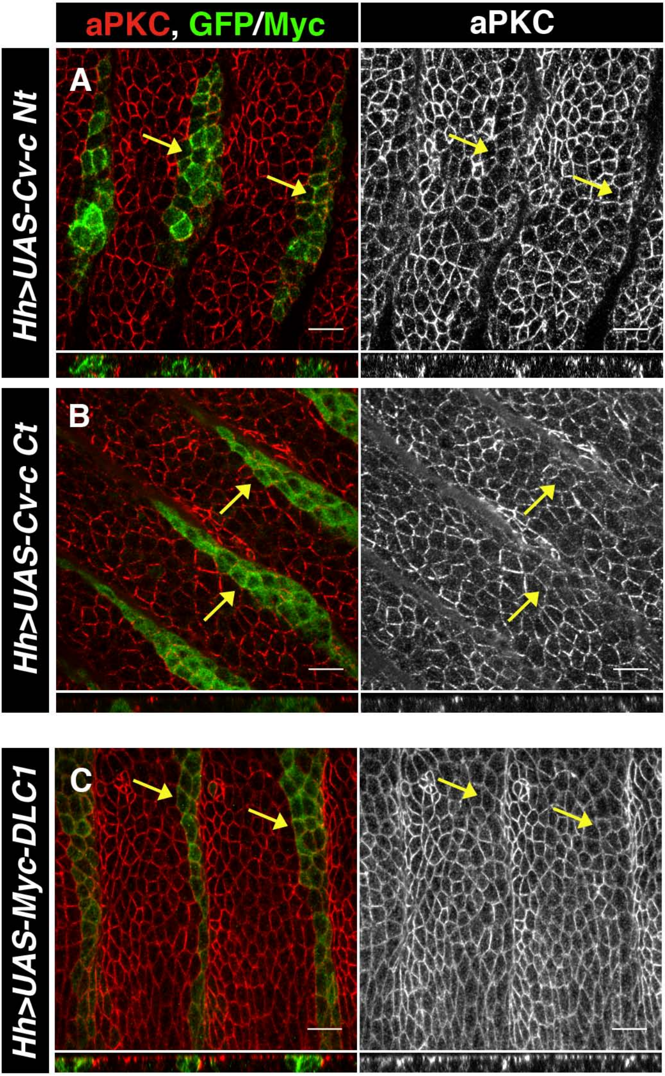

Supplementary Figure 1 **Sotillos et al.**

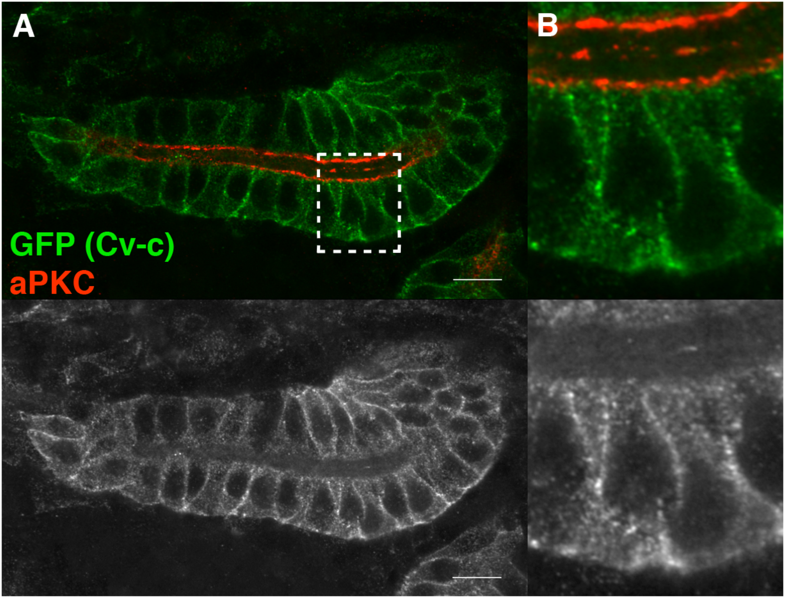

Supplementary Figure 2
Sotillos et al.

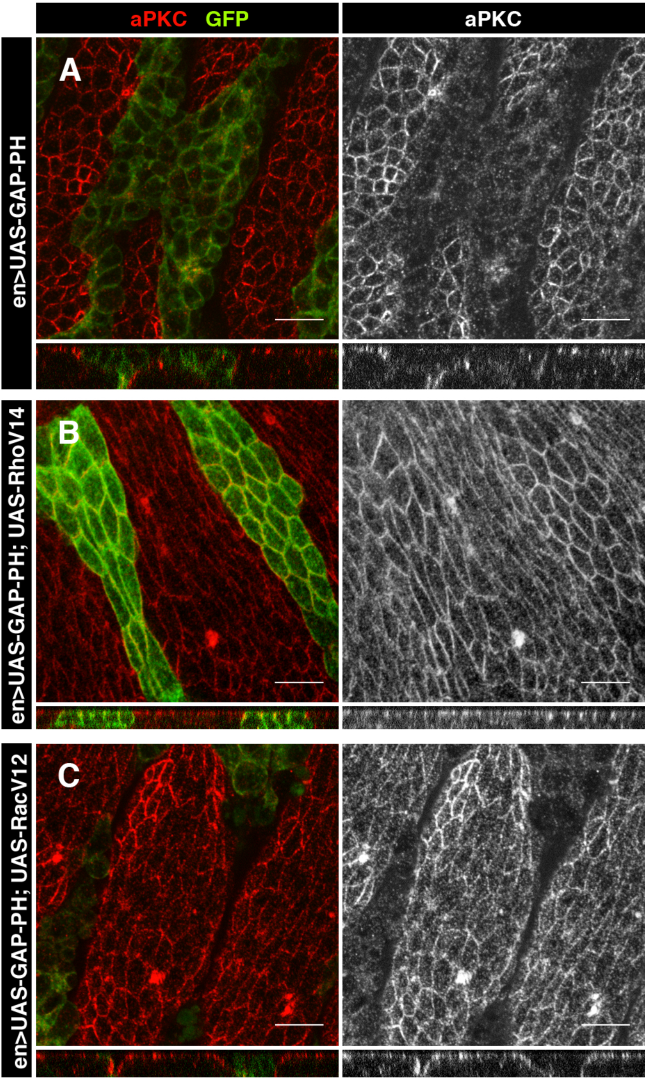

Supplementary Figure 3.

Sotillos et al.

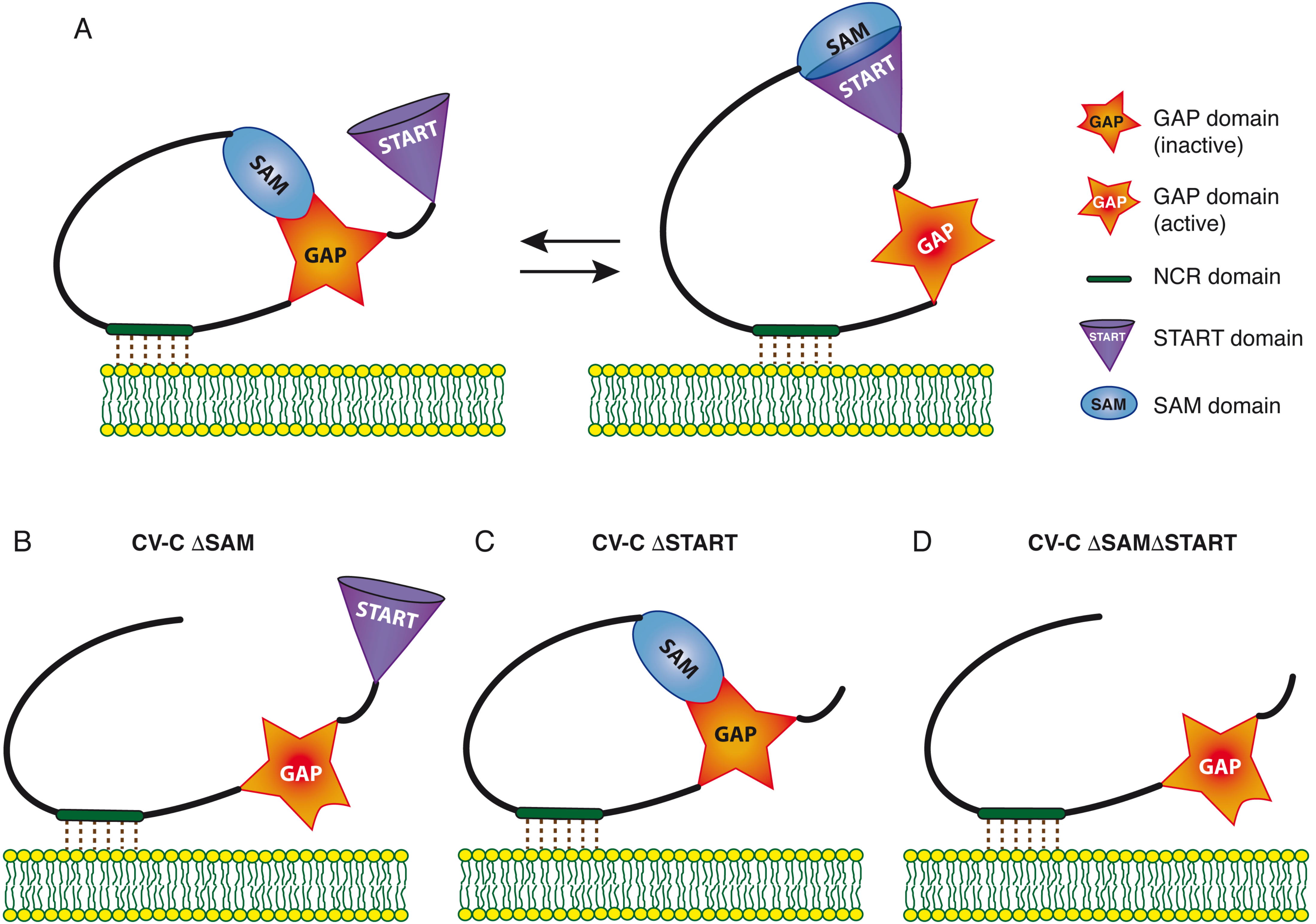

Supplementary Figure 4

Sotillos et al.

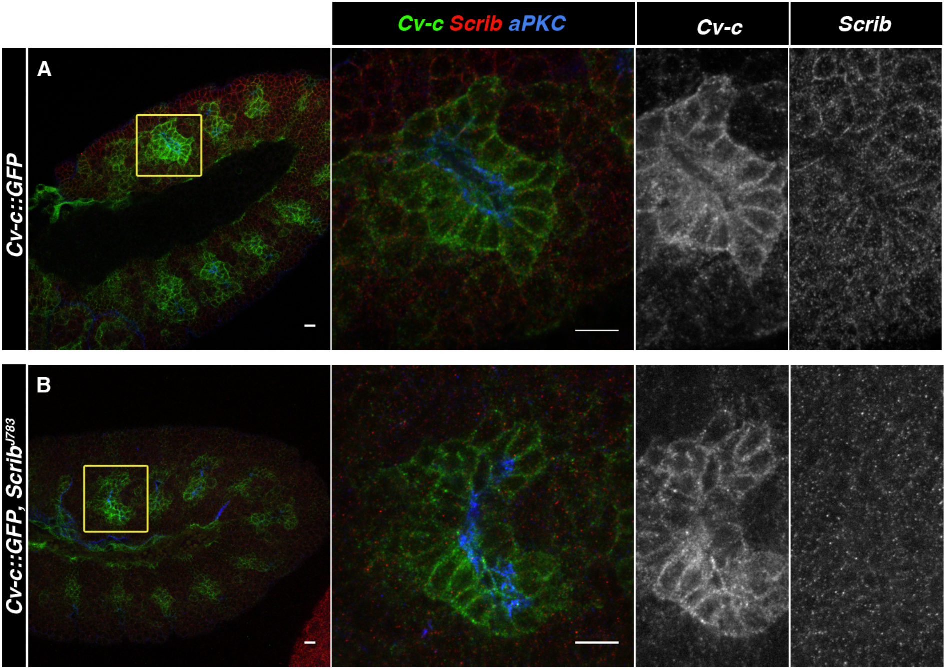

Supplementary Figure 5
Sotillos et al.
